# Incidence and Predictive Factors of Central Nervous System Dysfunction in Patients Consulting for Dengue Fever in Cayenne Hospital, French Guiana

**DOI:** 10.1371/journal.pone.0150828

**Published:** 2016-03-16

**Authors:** Félix Djossou, Guillaume Vesin, Bastien Bidaud, Emilie Mosnier, Christine Simonnet, Séverine Matheus, Christelle Prince, John Balcaen, Gerd Donutil, Gérald Egmann, Antoine Okandze, Denis Malvy, Mathieu Nacher

**Affiliations:** 1 Department of Infectious and Tropical Diseases, Centre Hospitalier de Cayenne, Cayenne, French Guiana; 2 Equipe d’Accueil EA3593 Ecosystèmes Amazoniens et Pathologie Tropicale, Université de Guyane, Cayenne, French Guiana; 3 Laboratory, Centre Hospitalier de Cayenne, Cayenne, French Guiana; 4 Laboratoire de virologie, Institut Pasteur de la Guyane, Cayenne, French Guiana; 5 Department of Pediatrics, Centre Hospitalier de Cayenne, Cayenne, French Guiana; 6 Service d’accueil des Urgences/Service d’aide médicale urgente, Centre Hospitalier de Cayenne, Cayenne, French Guiana; 7 Unité des Maladies Tropicales et du Voyageur, Centre Hospitalier Universitaire de Bordeaux, Bordeaux, France; 8 Centre d’Investigation Clinique Antilles Guyane, INSERM 1424, Centre Hospitalier de Cayenne, Cayenne, French Guiana; Institut Pasteur of Shanghai, CHINA

## Abstract

**Introduction:**

The frequency, the clinical characteristics, and the prognosis of dengue is highly variable. Dengue fever is associated with a range of neurological manifestations. The objective of the present study was to determine the incidence of neurological signs and their predictive factors using data from cases of dengue seen and followed in Cayenne Hospital during the Dengue 2 epidemic in 2013.

**Methods:**

In 2013, a longitudinal study using data from all cases of dengue seen in Cayenne hospital was collected. Medical records used a standardized form to collect demographic information, clinical signs and biological results and the date at which they were present. The analysis used Cox proportional modeling to obtain adjusted Hazard ratios.

**Results:**

A total of 1574 patients were included 221 of whom developed central nervous system signs. These signs were spontaneously resolutive. There were 9298person days of follow-up and the overall incidence rate for central nervous system signs was 2.37 per 100 person-days. The variables independently associated with central nervous system anomalies were headache, Adjusted Hazard ratio (AHR) = 1.9(95%CI = 1.4–2.6), bleeding AHR = 2 ((95%CI = 1.3–3.1), P = 0.001, abdominal pain AHR = 1.9 ((95%CI = 1.4–2.6), P<0.001, aches AHR = 2.1 ((95%CI = 1.5–2.9), P<0.001, and fatigue AHR = 1.5 ((95%CI = 1.3–1.7), P<0.001.

**Discussion:**

Overall, the present study suggests that neurological signs of dengue are not exceptional even in patients without the most severe features of dengue. These manifestations were spontaneously resolutive. Here it was not possible to distinguish between encephalitis or encephalopathy. Further studies would require more in depth exploration of the patients.

## Introduction

Dengue is the most frequent arbovirosis worldwide [1]. Although most symptomatic cases are self-limiting, a small proportion of cases will develop severe life-threatening forms of Dengue. The frequency, the clinical characteristics, and the prognosis of these severe forms of dengue is highly variable[2]. It may depend on the social environment of the territory where it occurs and the performance and accessibility of its health system. The local epidemiology, the circulating viruses also may influence the clinical spectrum of disease [3,4]. The definition of severe forms has been modified by WHO because, in practice, the former definition was difficult to use in all settings and did not adequately reflect the clinical polymorphism [2,5–9]. Such a broad definition aims at being more sensitive to avoid missing severe atypical cases. The Pathophysiology of severe dengue is still incompletely understood involving a mixture of host and viral factors [4]. Having a broad definition may thus be important for the clinicians but it may not be optimal to improve our understanding of the various forms of severity. The recent modifications of the WHO definition now include neurological manifestations which may directly result from the dengue virus or from indirect mechanisms such as liver dysfunction, bleeding, metabolic disorders. Dengue fever is associated with a range of neurological manifestations such as encephalopathy, encephalitis, immune-mediated syndromes, dengue muscle dysfunction, and neuro-ophtalmic disorders [10–15]. A recent review of neurological manifestations of dengue found that in South East Asia between 0.5% and 5.4% percent of patients had neurological manifestations, and that in Brazil this figure went up to 21% [15]. The involvement of the central nervous system has been described in all 3 classical types of dengue, dengue fever, dengue hemorrhagic fever, and dengue shock syndrome.

In French Guiana, a French overseas territory in South America, all four serotypes circulate with an increasing frequency and magnitude of epidemics. In the present study the objective was to determine the incidence of neurological signs and their predictive factors using longitudinal data from cases of dengue seen in Cayenne Hospital during the Dengue 2 epidemic in 2013.

## Methods

### Study type

In 2013, a study using data from all the cases of dengue seen in Cayenne hospital was collected.The patients were seen between 1 and 15 times between their first consultation and the end of the illness thus the study was longitudinal and allowed the calculation of incidence rates and survival analysis. The patients included for the calculation of incidence rates initially had no neurological manifestations.

### Context and health care organization

This was the third largest and the longest dengue epidemic to date in French Guiana. It was caused by a type 2 dengue virus. This was established by the national reference laboratory at Pasteur Institute in Cayenne which types viruses from a surveillance network of general private practitioners and cases from the hospital on the basis 1 virological typing for every 5 diagnoses. This aggregated data is used for epidemiologic surveillance of virus circulation and not for individual patient care. Thus 95% of circulating serotypes were DEN-2, less than 5% were DEN-4, and DEN-1 and DEN-3 were both below 1%. In order to prevent severe complications a proactive dengue circuit was set up in the emergency department and in the infectious diseases department. Patients suspected of dengue went through a special circuit with physicians collecting standardized information, systematically rehydrating patients intravenously and hospitalizing them if there was any warning sign or if the patients were isolated and unlikely to be able to be followed up. For Cayenne alone, there were an estimated 7231 cases, with 2672 probable or confirmed cases, 340 of whom were hospitalized (4.70%) 46 being severe (0.64%), with 2 deaths(2.7 per 10 000). The hospitalization rate was thus the highest to date. Patients that were not hospitalized were followed up every day at the infectious diseases outpatient department. The diagnosis of dengue was performed using NS1 tests, RTPCR or serology. Confirmed dengue was defined by positive NS1 or RTPCR, or seroconversion; probable dengue was defined as a single positive IgM serology result, or an evocative clinical presentation.

Medical records used a standardized data collection form to collect demographic information, clinical signs and biological results and the date at which they were present. A research nurse then retrospectively collected standardized anonymized information from the data collection form. The inclusion criteria for the present study were a final diagnosis of dengue by the senior infectiologist (FD) having reviewed each file. This included confirmed cases and suspected cases for which the epidemic context, the evocative clinical presentation and the negativity of other tests for differential diagnosis led to retain the diagnosis of dengue.

### Ethical and regulatory aspects

The data collection was part of the dengue emergency plan and was mandated by the regional health authorities. The anonymized monocentric data issued from medical records was analyzed which is authorized according to the Regulatory authorities (Commission NationaleInformatique et Libertésnumber TFN1490159N).

### Data analysis

Data analysis was performed using Stata 13. Quantitative variables were categorized according to statistical criteria using the 10th and 90th percentiles, the first and third quartiles and the median. The failure event was the first central nervous system dysfunction reported (defined as decreased Glasgow coma scale and or convulsions). Crude and stratum-specific incidence rates were calculated and single failure Cox proportional hazard models were used to identify predictors of neurological signs. To reduce the number of variables in a single model thematic models were build to select the variables for a final parsimonious multivariate model. The first model was built for clinical covariates, then a second one with biological covariates, finally the significant variables from both models were used in a third model. Schoenfeld and scaled Schoenfeld residuals were used to verify the proportionality of Hazards. The final model included variables that were significantly associated with the failure event in a single covariable analysis. The model was adjusted using a binary variable taking into account whether dengue was confirmed or probable.

## Results

### General results and incidence rate

A total of 1574 patients were included 221 of whom developed central nervous system signs (17 patients had central nervous system signs at their first consultation and thus were not included in the survival analysis). There were no details regarding whether these incident signs were linked to encephalopathy or encephalitis. There were 9298person-days of follow-up and the overall incidence rate for central nervous system signs was 2.37 per 100 person-days.Overall, after reviewing the files, 718 were probable dengue fever cases and 887 were biologically confirmed dengue either by NS1 (N = 742) and/or dengue-specific IgM seroconversion (N = 310).

Among patients, 50.5% were males and 49.5% were females; there were 60 children aged<1 year, 649 aged between 1 and 15 years, 843 aged between 16 and 65, and 29 aged over 65 years.

**Fig 1:**
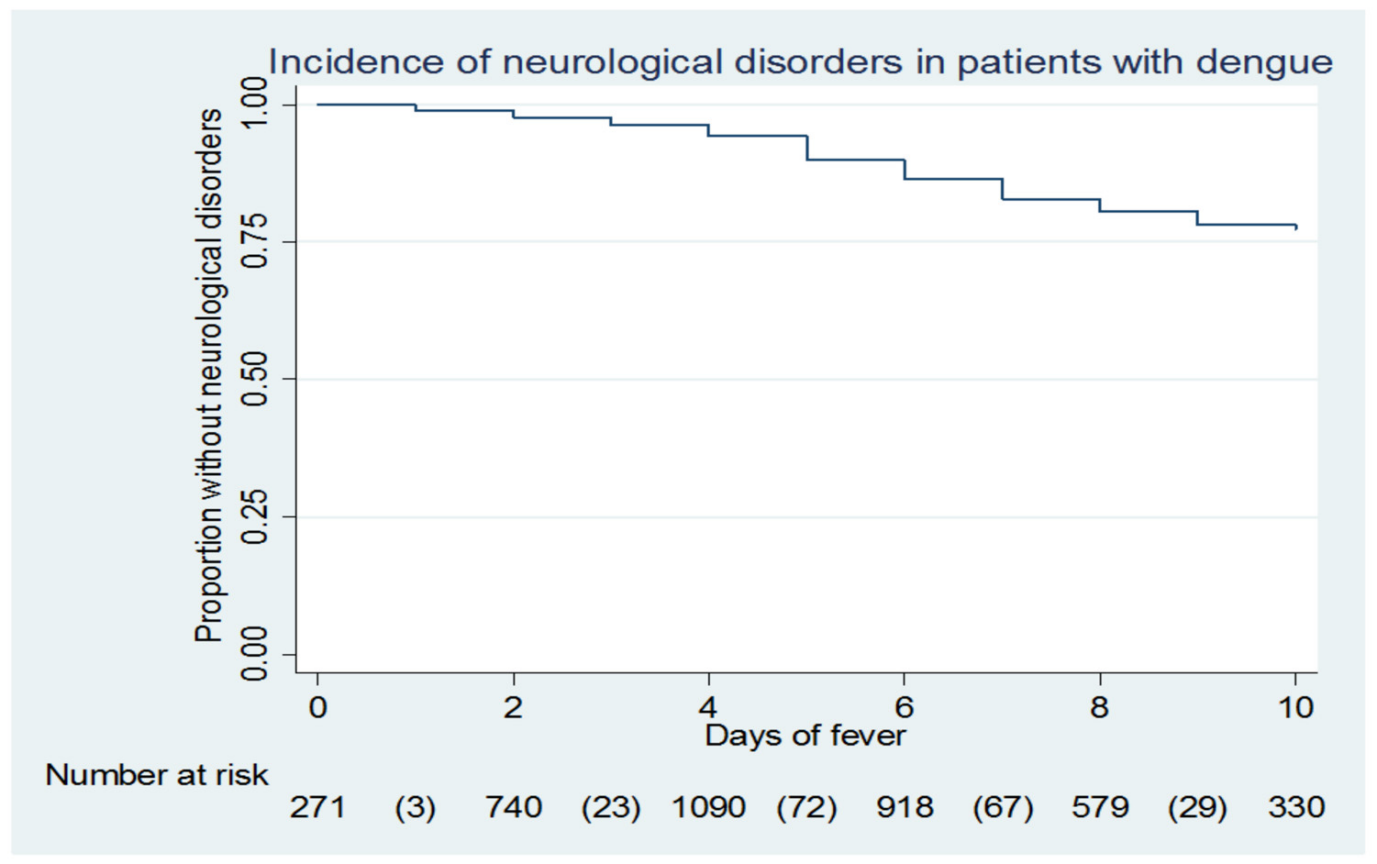
“Kaplan Meier curve for neurological disorder during dengue fever”.

**Fig 2:**
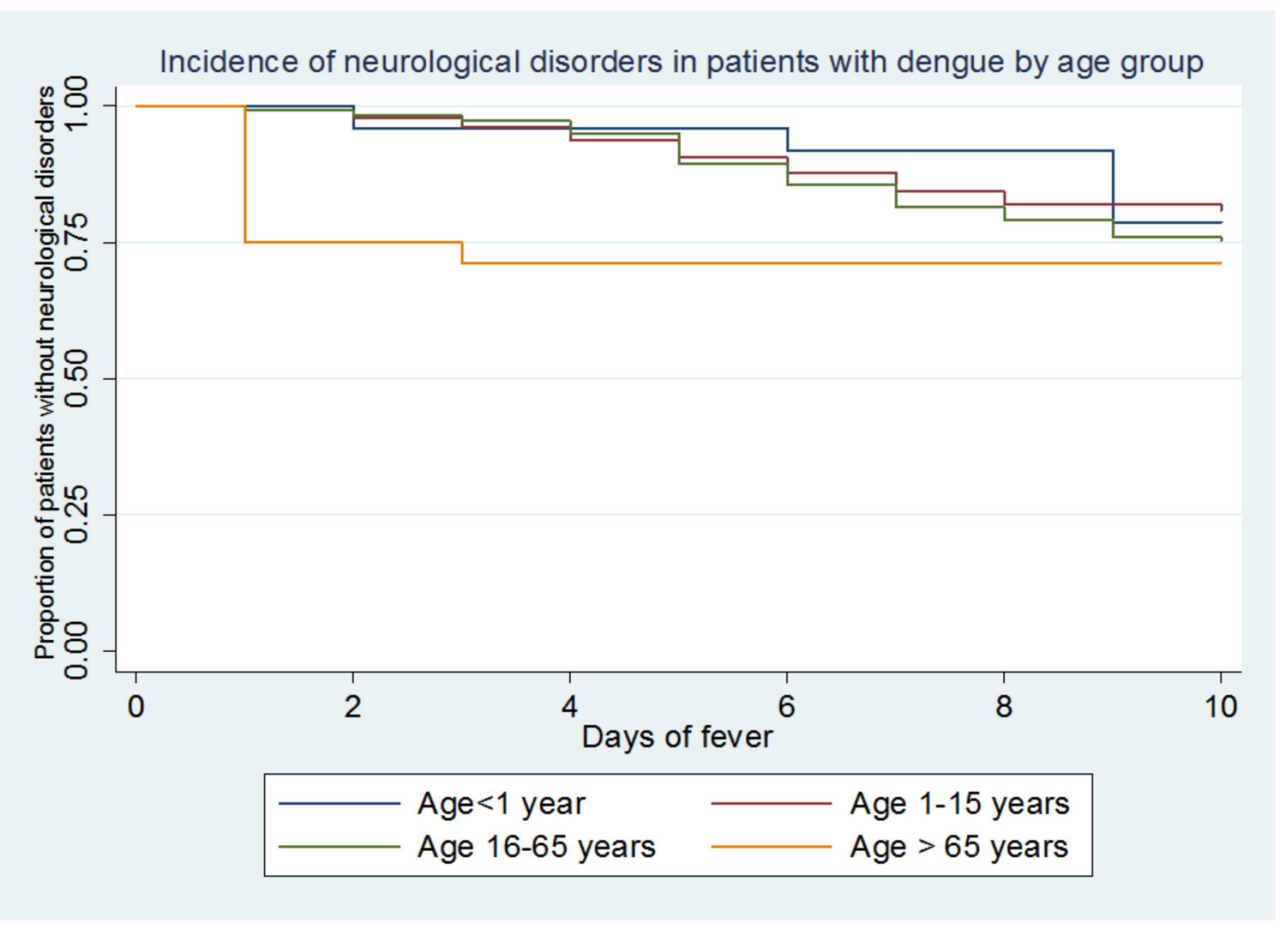
“Kaplan Meier curve for neurological disorder during dengue fever by age group”.

Fig 1 shows that over 20% of patients had CNS signs and that the highest incidence occurred between day 5 and day 6 of the fever. Fig 2 shows the incidence of CNS signs stratified by age, age groups <1 year and over 65 years had few patients (60 and 30, respectively).

### Clinical predictive factors

Table 1 shows the result of the model including clinical variables only. Table 1 shows that headaches, fatigue, malaise and lipothymia, aches, bleeding, and abdominal pain were significantly associated with CNS signs. The numbers reported represent the numbers of patients who, at some time had the sign. Therefore since a patient can initially have no given sign may then develop the sign, the totals for each variable exceed the total number of patients. The magnitude of the increased associated risk for these variables was moderate ranging between 1.5 and 2.2. Age and having a skin rash were not linked to CNS signs. For temperature, it seemed that having a moderate temperature elevation (between 37.5 and 38.4) was associated with a lower risk of CNS signs than temperatures below or above these values.

**Table 1:** Stratum specific incidence rates and adjusted hazard ratios for central nervous system signs for different clinical covariates.

Variables	N^†^	Incidence rate per 100 person-days	Crude hazard ratio (95% CI)	Adjusted hazard ratio* (95% CI), P
**Temperature (°C)**				
**<37.5**	1017	2.9	1	1
**37.5–38.4**	792	1.9	0.58 (0.4–0.86)	0.6 (0.4–0.9), P = 0.01
**38.5–41**	810	2	0.7 (0.4–1.05)	0.9 (0.6–1.3), P = 0.5
**Headaches**				
**No**	1124	1.3	1	1
**Yes**	1034	4.2	3.1 (2.3–4.1)	2 (1.4–2.7), P<0.001
**Aches/myalgia/joint pain**				
**No**	1149	1.2	1	1
**Yes**	919	4.7	3.7 (2.8–5)	2.2 (1.6–3.1), P<0.001
**Nose/Gum bleeding or Meno-metrorrhagia**				
**No**	1544	2.2	1	1
**Yes**	250	7	2.6 (1.7–3.9)	2.1 (1.4–3.2), P<0.001
**Petechia**				
**No**	1538	2.2	1	1
**Yes**	249	5.1	2 (1.4–2.9)	1.5 (1.07–2.2), P = 0.02
**Fatigue/malaise/Syncope**				
**No**	1297	0.6	1	1
**Yes**	994	4.7	1.5 (1.4–1.7)	1.5 (1.3–1.7), P<0.001
**Rash**				
**No**	1504	2.4	1	1
**Yes**	266	2.7	1 (0.7–1.5)	0.8 (0.5–1.2), P = 0.2
**Abdominal pain**				
**No**	1443	1.9	1	1
**Yes**	510	5.6	2.5 (1.9–3.3)	1.9 (1.4–2.5), P<0.001
**Age (years)**				
**<1**	60	1.4	1	1
**1–15**	649	2	1.5 (0.5–4.8)	0.6(0.2–2), P = 0.4
**16–65**	843	2.6	2 (0.65–6.5)	0.6(0.2–2), P = 0.4
**>65**	29	1.3	1 (0.2–4.9)	0.4 (0.08–2.3), P = 0.3

**†** *Obtained using the stratified incidence data*. *Some patients may go from one category to another during follow up visits (for example from no bleeding signs to bleeding signs) thus N may be often significantly greater than the total number of patients*.

*Obtained using a Cox proportional hazard model, *using schoenfeld and scaled schoenfeld residuals to verify the hazard proportionality*

### Biological predictive factors

Table 2 shows the model with the biological variables only. Table 2 shows that higher serum protein concentrations, and lower lymphocyte counts were significantly associated with CNS signs. Creatinine and sodium concentration, neutrophils and platelet counts were not significantly associated with central nervous system dysfunction and were not retained in the parsimonious model.

**Table 2:** Stratum specific incidence rates and adjusted hazard ratios for central nervous system signs for different significant biological covariates.

Variables	N^†^	Incidence rate per 100 person-days	Crude hazard ratio (95% CI)	Adjusted hazard ratio* (95% CI), P
**Hematocrit (%)**				
**<37.2**	628	1.6	1	1
**[37.3–40.4[**	575	2.3	2.4 (1.05–2.7)	1.3 (0.9–2), P = 0.16
**[40.4–44.1]**	593	2.3	2.7 (1.3–3.2)	1.3 (0.9–2), P = 0.12
**>44.1**	447	3	3 (1.08–2.7)	1.3 (0.8–2), P = 0.18
**Lymphocyte count (G/L)**				
**<1.4**	694	3.1	2.14 (1.4–3.2)	1.8 (1.2–2.9), P = 0.007
**[1.4–2.3[**	718	2.8	1.6 (1.03–2.4)	1.4 (0.9–2.2), P = 0.1
**[2.3–3.8]**	615	2.2	1.3(0.9–2)	1.3 (0.8–1.9), P = 0.2
**>3.8**	539	1.6	1	1
**Proteinemia (g/L)**				
**<67**	369	1.6	1	1
**[67–72[**	594	3.1	2.3(1.5–3.3)	2.2 (1.5–3.2), P<0.001
**[72–77]**	773	2.3	1.6 (1.06–2.5)	1.6 (1–2.5), P = 0.04
**>77**	646	2.6	2.1 (1.4–3)	2 (1.3–2.9), P<0.001

*† Obtained using the stratified incidence data*. *Some patients may go from one category to another during follow up visits (for example from no hypoproteinemia to low proteinemia)*.*Thus N may be often significantly greater than the total number of patients*.

*Obtained using a Cox proportional hazard model, *using schoenfeld and scaled schoenfeld residuals to verify the hazard proportionality*

### Clinical and biological predictive factors

Table 3 shows that in the final model including both significant clinical and biological variables, the clinical variables in Table 1 were still significantly associated with the outcome but that for the biological variables identified in Table 2 most of the significant associations disappeared, and that the significance of the association between proteinemia in the second quartile and CNS signs was not clear. Thus clinical variables were more reliable at predicting CNS anomalies than biological variables. The adjustment using the probable/confirmed dengue was not significantly associated with the outcome (P = 0.25) and did not change the findings. It was thus discarded from the final model. Models without purpura (which was presumed to be correlated to hemorrhage) and headaches (a common non-specific sign of dengue) were attempted but the AIC (Akaike’s Information criterion) showed that they were less informative than models with these variables which were thus retained. Overall, headaches, fatigue, malaise and lipothymia, aches, bleeding, and abdominal pain were significantly associated with an increased risk of CNS signs.

**Table 3:** Stratum specific incidence rates and adjusted hazard ratios for central nervous system signs for different significant clinical and biological covariates.

Variables	Adjusted hazard ratio* (95% CI), P
**Lymphocyte count (G/L)**	
**<1.4**	1.4 (0.9–2.2), P = 0.14
**[1.4–2.3[**	1.3 (0.8–2), P = 0.2
**[2.3–3.8]**	1.1 (0.7–1.7), P = 0.5
**>3.8**	1
**Proteinemia (g/L)**	
**<67**	1
**[67–72[**	1.7 (1.1–2.5), P = 0.007
**[72–77]**	1.1 (0.7–1.8), P = 0.5
**>77**	1.4 (0.9–2), P = 0.1
**Headaches**	
**No**	1
**Yes**	1.9 (1.4–2.6), P<0.001
**Aches/myalgia/joint pain**	
**No**	1
**Yes**	2.1 (1.5–2.9), P<0.001
**Nose/Gum bleeding or Meno-metrorrhagia**	
**No**	1
**Yes**	2 (1.3–3.1), P = 0.001
**Petechia**	
**No**	1
**Yes**	1.3 (0.9–1.9), P = 0.13
**Fatigue/malaise/Syncope**	
**No**	1
**Yes**	1.5 (1.3–1.7), P<0.001
**Abdominal pain**	
**No**	1
**Yes**	1.9 (1.4–2.6), P<0.001
**Temperature (°C)**	
**<37.5**	1
**37.5–38.4**	0.5 (0.3–0.8), P = 003
**38.5–41**	0.8(0.5–1.3), P = 0.3

## Discussion

Here in a small city with easy access to emergency care, quality care and a policy of hospitalizing patients with any clinical, biological or social warning sign, over 20% of the patients seen for dengue fever had central nervous system dysfunction mostly around the 5th or 6th day of fever. This proportion is much higher than in the studies in South East Asia [16–19] but similar to a study in Brazil [20]. The study in Brazil recruited patients admitted to a tertiary care whereas our study included all patients seen at the hospital, both outpatients consulting an infectiologist and hospitalized patients. Given the non specific nature of the data collection form, it was difficult to know whether CNS signs were linked to encephalopathy or encephalitis, or if these signs reflected general exhaustion. Most patients did not have CSF examination because thrombocytopenia was assumed to make the spinal tap risky. All these clinical anomalies of unknown pathophysiology were spontaneously resolutive.

The incidence of CNS dysfunction was statistically linked to Headaches, fatigue, malaise and fainting, aches, bleeding, and abdominal pain. Headaches may have been preceding more patent cerebral dysfunction. Bleeding manifestations and signs that may have reflected hypovolemia could alter the central nervous system's function. Variables that have been reported to be associated with encephalopathy such as liver enzymes, sodium concentration were not significantly linked to CNS dysfunction. Patients on admission receive prompt intraveinous rehydration, thus avoiding in many cases the severe complications of dengue. Here, the incidence rate of CNS dysfunction of 2.37 per 100 person-days assumed that hospitalized patients were initially neurologically not symptomatic. Thus 17 patients that arrived with such signs on admission were not counted in the incidence rate calculation.

A review [15] of neurological disorders during dengue fever reports the frequent overlap between encephalopathy and encephalitis and immune mediated syndromes, and the absence of a standardized definition of encephalitis or encephalopathy. It is also reported that in most studies cerebrospinal fluid testing for dengue virus or dengue-specific IgM or NS1 antigen testing are not consistently performed, as was the case in French Guiana. Apart from the neurological manifestations themselves, the studies on neurological manifestations of dengue also report various degrees of underlying severity with some reporting severe dengue in most cases [19], whereas some showed a prominent neurological aspect[11]. The reported case fatality rates are often high, notably when encephalopathy reflects the severity of dengue [15] but the study from Brazil [20] found that the presence of CNS involvement did not influence the prognosis of dengue.

Neurological manifestations of dengue generally require supportive care, airway maintenance, and correction of fluid and electrolyte balance when possible [15]. In contrast with many studies, in the present study, no fatality was reported among those with CNS involvement. Given the prompt rehydration and the specific dengue follow up set up during the epidemic much of the severity may have been averted. Also the definition of neurological dysfunction may have varied between studies.

The present epidemic was caused by a Dengue 2 virus. This was based on aggregated data from the reference center for the epidemiologic surveillance of arboviruses, and the data was not connected to individual patients. Since 95% of the viruses were DEN-2 it seems plausible that most patients with CNS dysfunction had DEN-2 viruses. However, it is not possible to rule out that certain serotypes were overrepresented in patients with CNS dysfunction [21]. It is not known whether other viruses have similar consequences on neurological signs, either through varying degrees of neurotropism or differences in virulence. Thus, the incidence and the predictive factors of CNS anomalies should be assessed in epidemics caused by different viruses. Furthermore, prior infection by different dengue viruses may also have influenced the incidence of CNS anomalies in those concerned because of facilitating antibodies. However, this hypothesis should be tested formally. In a study in Brazil, including patients with encephalopathy or encephalitis, it was suggested that the pathogenesis of neurological involvement was distinct from the pathogenesis of dengue hemorrhagic fever [20]. The number of patients involved and the mix of encephalitis and encephalopathy may however have been insufficient to conclude regarding pathogenesis, notably for encephalopathy in cases of dengue hemorrhagic fever [19,22]. Thus while encephalitis may be linked to neurotropism, encephalopathy seems more related to overall severity

This is the first large longitudinal study to provide incidence rates for CNS signs during dengue fever among patients seen at a reference hospital. Overall, the present study suggests that neurological signs of dengue are not exceptional even in patients without the most severe features of dengue. These manifestations were spontaneously resolutive and were associated with patients various algic or hemorrhagic manifestations. Given the coarse clinical definition of the studied outcome and the lack of cerebral spinal fluid data further studies should better define the neurologic syndromes and document the nature of the CNS anomalies to understand the pathogenesis of different manifestations and their respective frequency [15].

## References

[1] Bhatt S, Gething PW, Brady OJ, Messina JP, Farlow AW, Moyes CL, et al (2013) The global distribution and burden of dengue. Nature 496: 504–507. 10.1038/nature12060 23563266 PMC3651993

[2] Deen JL, Harris E, Wills B, Balmaseda A, Hammond SN, Rocha C, et al (2006) The WHO dengue classification and case definitions: time for a reassessment. Lancet 368: 170–173. 16829301 10.1016/S0140-6736(06)69006-5

[3] Rothman AL, Ennis FA (1999) Immunopathogenesis of Dengue hemorrhagic fever. Virology 257: 1–6. 10208914 10.1006/viro.1999.9656

[4] Soundravally R, Hoti SL (2007) Immunopathogenesis of dengue hemorrhagic fever and shock syndrome: role of TAP and HPA gene polymorphism. Hum Immunol 68: 973–979. 10.1016/j.humimm.2007.09.007 18191725

[5] Narvaez F, Gutierrez G, Perez MA, Elizondo D, Nunez A, Balmaseda A, et al (2011) Evaluation of the traditional and revised WHO classifications of Dengue disease severity. PLoS Negl Trop Dis 5: e1397 10.1371/journal.pntd.0001397 22087348 PMC3210746

[6] Bandyopadhyay S, Lum LC, Kroeger A (2006) Classifying dengue: a review of the difficulties in using the WHO case classification for dengue haemorrhagic fever. Trop Med Int Health 11: 1238–1255. 16903887 10.1111/j.1365-3156.2006.01678.x

[7] Rigau-Perez JG (2006) Severe dengue: the need for new case definitions. Lancet Infect Dis 6: 297–302. 16631550 10.1016/S1473-3099(06)70465-0

[8] van de Weg CA, van Gorp EC, Supriatna M, Soemantri A, Osterhaus AD, Martina BE. (2012) Evaluation of the 2009 WHO dengue case classification in an Indonesian pediatric cohort. Am J Trop Med Hyg 86: 166–170. 10.4269/ajtmh.2012.11-0491 22232468 PMC3247126

[9] Thangaratham PS, Tyagi BK (2007) Indian perspective on the need for new case definitions of severe dengue. Lancet Infect Dis 7: 81–82. 17251078 10.1016/S1473-3099(07)70005-1

[10] Verma R, Sahu R, Holla V (2014) Neurological manifestations of dengue infection: a review. J Neurol Sci 346: 26–34. 10.1016/j.jns.2014.08.044 25220113

[11] Solomon T, Dung NM, Vaughn DW, Kneen R, Thao LT, Raengsakulrach B, et al (2000) Neurological manifestations of dengue infection. Lancet 355: 1053–1059. 10744091 10.1016/S0140-6736(00)02036-5

[12] Solbrig MV, Perng GC (2015) Current neurological observations and complications of dengue virus infection. Curr Neurol Neurosci Rep 15: 550. 10.1007/s11910-015-0550-4 25877545

[13] Soares CN, Faria LC, Peralta JM, de Freitas MR, Puccioni-Sohler M (2006) Dengue infection: neurological manifestations and cerebrospinal fluid (CSF) analysis. J Neurol Sci 249: 19–24. 16870213 10.1016/j.jns.2006.05.068

[14] Misra UK, Kalita J, Syam UK, Dhole TN (2006) Neurological manifestations of dengue virus infection. J Neurol Sci 244: 117–122. 16524594 10.1016/j.jns.2006.01.011

[15] Carod-Artal FJ, Wichmann O, Farrar J, Gascon J (2013) Neurological complications of dengue virus infection. Lancet Neurol 12: 906–919. 10.1016/S1474-4422(13)70150-9 23948177

[16] Cam BV, Fonsmark L, Hue NB, Phuong NT, Poulsen A, Heegaard ED. (2001) Prospective case-control study of encephalopathy in children with dengue hemorrhagic fever. Am J Trop Med Hyg 65: 848–851. 11791985 10.4269/ajtmh.2001.65.848

[17] Pancharoen C, Thisyakorn U (2001) Neurological manifestations in dengue patients. Southeast Asian J Trop Med Public Health 32: 341–345. 11556587

[18] Thisyakorn U, Thisyakorn C, Limpitikul W, Nisalak A (1999) Dengue infection with central nervous system manifestations. Southeast Asian J Trop Med Public Health 30: 504–506. 10774659

[19] Hendarto SK, Hadinegoro SR (1992) Dengue encephalopathy. Acta Paediatr Jpn 34: 350–357. 1509881 10.1111/j.1442-200x.1992.tb00971.x

[20] Domingues RB, Kuster GW, Onuki-Castro FL, Souza VA, Levi JE, Pannuti CS. (2008) Involvement of the central nervous system in patients with dengue virus infection. J Neurol Sci 267: 36–40. 17959198 10.1016/j.jns.2007.09.040

[21] Bordignon J, Strottmann DM, Mosimann AL, Probst CM, Stella V, Noronha L. (2007) Dengue neurovirulence in mice: identification of molecular signatures in the E and NS3 helicase domains. J Med Virol 79: 1506–1517. 17705192 10.1002/jmv.20958

[22] Malavige GN, Ranatunga PK, Jayaratne SD, Wijesiriwardana B, Seneviratne SL, Karunatilaka DH. (2007) Dengue viral infections as a cause of encephalopathy. Indian J Med Microbiol 25: 143–145. 17582186 10.4103/0255-0857.32722

